# A Worldwide Analysis of Beta-Defensin Copy Number Variation Suggests Recent Selection of a High-Expressing *DEFB103* Gene Copy in East Asia

**DOI:** 10.1002/humu.21491

**Published:** 2011-03-08

**Authors:** Robert J Hardwick, Lee R Machado, Luciana W Zuccherato, Suzanne Antolinos, Yali Xue, Nyambura Shawa, Robert H Gilman, Lilia Cabrera, Douglas E Berg, Chris Tyler-Smith, Paul Kelly, Eduardo Tarazona-Santos, Edward J Hollox

**Affiliations:** 1 Department of Genetics, University of LeicesterLeicester, United Kingdom; 2 Departamento de Biologia Geral, Instituto de Ciências Biológicas, Universidade Federal de Minas GeraisBelo Horizonte, Brazil; 3 Wellcome Trust Sanger InstituteHinxton, United Kingdom; 4 Tropical Gastroenterology and Nutrition Group, University of Zambia School of MedicineLusaka, Zambia; 5 Institute of Cell and Molecular Science, Barts and The London School of Medicine, Queen Mary University of LondonLondon, United Kingdom; 6 Johns Hopkins Bloomberg School of Public Health, Johns Hopkins UniversityBaltimore, Maryland; 7 Ascociación Benéfica PRISMALima, Peru; 8 Department of Molecular Microbiology, Washington University School of MedicineSt. Louis, Missouri

**Keywords:** CNV, defensin, antimicrobial, influenza, paralogue ratio test

## Abstract

Beta-defensins are a family of multifunctional genes with roles in defense against pathogens, reproduction, and pigmentation. In humans, six beta-defensin genes are clustered in a repeated region which is copy-number variable (CNV) as a block, with a diploid copy number between 1 and 12. The role in host defense makes the evolutionary history of this CNV particularly interesting, because morbidity due to infectious disease is likely to have been an important selective force in human evolution, and to have varied between geographical locations. Here, we show CNV of the beta-defensin region in chimpanzees, and identify a beta-defensin block in the human lineage that contains rapidly evolving noncoding regulatory sequences. We also show that variation at one of these rapidly evolving sequences affects expression levels and cytokine responsiveness of *DEFB103*, a key inhibitor of influenza virus fusion at the cell surface. A worldwide analysis of beta-defensin CNV in 67 populations shows an unusually high frequency of high-*DEFB103*-expressing copies in East Asia, the geographical origin of historical and modern influenza epidemics, possibly as a result of selection for increased resistance to influenza in this region. Hum Mutat 32:743–750, 2011. © 2011 Wiley-Liss, Inc.

## Introduction

Copy number variation (CNV) is an important source of human genetic diversity, and can result in phenotypic diversity and different susceptibilities to disease [Conrad et al., 2009; Sudmant et al., 2010; Wain et al., 2009]. Most studies have been based either on genomewide surveys where CNV detection and calling is heavily biased toward copy number changes that cause a large change in dosage, such as deletions, or on locus-specific studies on limited sample sets with error-prone calling of diploid copy number. Genome-wide analyses suggest overall reduced purifying selection on gene copy number in *Drosophila melanogaster* [Dopman and Hartl, 2007; Emerson et al., 2008], and in humans [Nguyen et al., 2008], yet analyses of individual CNVs have suggested neutral evolution, for example, at those containing chemosensory genes [Nozawa et al., 2007; Young et al., 2008]. There is also some evidence for positive selection acting on certain CNVs in humans, such as the amylase locus where populations with a higher average copy number have a history of starch-rich diets [Perry et al., 2007], and the *UGT2B17* gene (MIM♯ 601903), which encodes a minor histocompatibility antigen [Xue et al., 2008].

Beta-defensins are a family of proteins characterized by a conserved 6-cysteine motif, which results in a characteristic arrangement of three disulfide bridges in the mature protein [Ganz, 2003; Pazgier et al., 2006]. Six members of the beta-defensin family at chromosome 8p23.1 (*DEFB4*, *DEFB103*, *DEFB104*, *DEFB105*, *DEFB106*, *DEFB107*) are annotated as a block that is copy number variable, with diploid copy numbers between 1 and 12 [Groth et al., 2008; Hollox et al., 2003, 2005, 2008a]. We refer to this copy number variable block as the beta-defensin CNV. Three other beta-defensin genes (*DEFB1*, *DEFB108,* and *DEFB109*) are also annotated to this region but lie outside the beta-defensin CNV; *DEFB1* is not copy number variable, whereas *DEFB108* and *DEFB109* show very complex structural variation within olfactory receptor regions, with an unclear relationship to the beta-defensin CNV. All these beta-defensin genes encode short cationic peptides between 2 and 6 kDa in size and variously expressed in epithelia and the testis. *DEFB4* (MIM♯ 602215) and *DEFB103* (MIM♯ 606611) are the best-characterized beta-defensins, showing antimicrobial properties and pro- and anti-inflammatory signaling capacity through interactions with several receptors, including CCR6 and MCR1 [Candille et al., 2007; Feng et al., 2006; Harder et al., 2001; Niyonsaba et al., 2004; Schibli et al., 2002; Semple et al., 2010; Yang et al., 1999].

The beta-defensin CNV is at least 250 kb in size and present in multiple copies at two loci that are separated by ∼5 Mb of single copy sequence, which itself is polymorphically inverted [Abu Bakar et al., 2009; Giglio et al., 2001; Sugawara et al., 2003]. Increased copy number is correlated with increased levels of hBD-2 peptide (encoded by the *DEFB4* gene) and has been associated with an increased risk of developing the inflammatory skin disease psoriasis [Hollox et al., 2008c; Jansen et al., 2009]. This variation accounts for about 5% of the total sibling recurrence risk of this disease, equivalent to the amount of sibling recurrence risk identified in the first generation of genomewide association studies [Roberson and Bowcock, 2010]. Initial studies of association of beta-defensin CNV with Crohn's disease have not been substantiated in larger studies, and demonstrate the importance of accurate copy number typing in association studies [Aldhous et al., 2010; Bentley et al., 2009; Fellermann et al., 2006]. The beta-defensin CNV has a high copy number mutation rate of around 0.7% per generation, caused mostly by allelic recombination in the 5 Mb between the two CNV loci leading to the inheritance of novel combinations of blocks [Abu Bakar et al., 2009]. The beta-defensin genes in this CNV do not show strong evidence of positive selection at the amino acid level [Hollox and Armour, 2008], but such selection would be distinct from selection for gene dosage variation, and this remains unstudied. Indeed, it could be argued that selection for gene dosage changes would prevent coding sequence divergence between paralogues, as the favorable trait is increased or decreased levels of the same protein [Kondrashov et al., 2002; Schuster-Bockler et al., 2010].

Here, we investigate the recent evolution of the beta-defensin CNV by showing beta-defensin copy number variation in chimpanzees, identifying two types of beta-defensin CNV block by comparative sequence analysis, and showing that regulatory sequences have rapidly diverged. We analyse the frequency of the two types of beta-defensin CNV block in 67 populations from across the globe, and discuss possible interpretations of the pattern seen.

## Materials and Methods

### Samples

Unrelated human DNA samples were from the standardized subset of 1,056 individuals from the HGDP-CEPH panel [Cann et al., 2002; Rosenberg, 2006], the HapMap phase I panel (45 CHB—Chinese from Beijing, 45 JPT—Japanese from Tokyo, 60 CEU—European Americans from Utah, 60 YRI—Yoruba from Nigeria), the Health Protection Agency or directly extracted from human peripheral blood (Supp. Table S1). *Pan troglodytes troglodytes* DNA samples were from mouthswabs self-administered by animals, and extracted using a column-based method, according to the manufacturer's instructions (Isohelix, Maidstone, UK). *Pan troglodytes verus* DNA samples were from lymphoblastoid cell lines made available by the INPRIMAT consortium. Human DNA samples used in this study were either from publically available cell lines maintained by Coriell Cell Repositories or the Health Protection Agency (UK) or from peripheral blood collected under appropriate local ethical consent.

### Copy Number Typing in Humans

Beta-defensin copy number was directly determined using a triplex paralogue ratio test (PRT), as previously published [Aldhous et al., 2010] (Supp. Fig. S1). The quality of copy number calling can be judged qualitatively by examining histograms of raw copy number calls because, given the expectation that copy numbers are integers, values will cluster about those integer values. The raw data, displayed as a mean of the triplex assay, are shown on a histogram (Supp. Fig. S2a and b), and show clear clustering around integer values. The three independent measures of copy number produced by this test were used to infer the most likely copy number for each sample using a maximum-likelihood approach [Aldhous et al., 2010; Hollox et al., 2008b]. This approach also allows, for each sample, a statistical estimate of the confidence in the calling of that particular copy number compared to all other potential copy numbers. Each copy number call was assigned a *P*-value, representing the likelihood of that copy number call compared to all other copy number calls, with 95.5% of samples having copy number calls with *P*<0.05.

The copy number of clade II was determined using polymerase chain reaction (PCR) amplification followed by restriction enzyme digestion and quantification of the two digested products. Primers flanking rs2737902 (5′-CCCAGAACTAACACACCCTTG-3′ and 5′-CTCTTGGCTCAGGAGCATTC-3′) were used to amplify a PCR product from 10 ng genomic DNA, following the standard Kapa *Taq* DNA polymerase reaction conditions (Kapa Biosystems) and cycling conditions of at 98°C 2 min, followed by 27 cycles of 98°C 20 sec, 63°C 60 sec, 70°C 60 sec, and a final extension cycle of 70°C for 10 min. A total of 2 µl of PCR product was added to a restriction enzyme digestion mix containing 1 unit of *Bsr*I (New England Biolabs, Beverly, MA), incubated at 37°C for at least 4 hr and then electrophoresed; fragments were quantified using an ABI3130 genetic analyzer and GeneMapper software (Applied Biosystems, Bedford, MA). Known clade II copy number controls were included in every experiment, and used to calibrate the raw ratio results, to minimize the effect of heteroduplex formation, which consistently underestimates the amount of cut fragment by being refractory to restriction enzyme digestion. Copy number of clade II was determined using a maximum-likelihood approach given the known total beta-defensin copy number and the ratio of cut:uncut fragments.

### Copy Number Typing in Chimpanzees

Two approaches were used to determine the copy number and size of the variable beta-defensin region in the chimpanzees (Supp. Fig. S1). The HSPD21 PRT assay described previously [Aldhous et al., 2010] was predicted to work with chimpanzee DNA, based on sequence comparison. In addition, analysis of the NCBI sequence trace archive identified a chimpanzee-specific variable short tandem repeat within the putative beta-defensin copy number variable region (Supp. Fig. S3). The short tandem repeat was amplified using final concentrations of 1 µM of each primer (5′-FAM-GGCTCTCACTTGGTCTCTGG-3′, 5′-GAGTGTTTGCTGAGGCTTCC-3′), 3 mM of each dNTP, 1 × Kappa HiFi buffer (containing 2 mM MgCl_2_), 0.2 units Kappa HiFi HotStart *Taq* DNA Polymerase (Kappa Biosystems, Woburn, MA), and 10 ng genomic DNA in a final volume of 10 µl. Thermal cycling was performed on a Perkin-Elmer 9700 (Norwalk, CT), at 95°C 2 min, followed by 30 cycles of 95°C 30 sec, 58°C 30 sec, 70°C 30 sec. Of the 15 samples typed using both assays, 12 reported copy number variation in agreement with each other, suggesting that the region between *DEFB4* and *SPAG11*, at least, varies in copy number *en bloc*, at least on most chromosomes. We cannot rule out heterogeneity in copy number of some blocks, but this heterogeneity was seen on samples showing wide 95% confidence intervals for the PRT, and suggests that PRT assays thoroughly optimized for the chimpanzee genome are likely to show smaller confidence intervals.

### Paralogue-Specific PCR and Sequencing

Potential nucleotide sites variable between paralogues in chimpanzees were identified by searching the Trace Archive at NCBI. Following identification of a *Fok*I RFLP near *DEFB4* that showed a very high rate of apparent heterozygosity in our chimpanzee samples (indicating that it was likely a variant distinguishing paralogues), a paralogue-specific PCR was designed to specifically amplify two copies of each paralogue from four copy *Pan troglodytes verus* samples. The paralogue specific primer was 5′-TGCTTTGCTCCTCCTGCA-3′ or 5′-TGCTTTGCTCCTCCTGCG-3′, with a common reverse primer 5′-AGTTGCTCACATATGAGAGC-3′. Amplification used Kapa HiFi HotStart *Taq* DNA polymerase according to the manufacturer's instructions. The PCR products were directly sequenced and, because no sequence showed apparent heterozygosity at more than one position, sequence haplotypes could be assigned.

### Statistical Analysis

Summary statistic calculations and analysis of variance were performed using Microsoft Excel. Principal component analysis and biplot generation was performed using R, on 54 populations where the number of samples was greater than 12. Mapping was performed using Surfer 8.0 (Golden Software, Golden, CO), F_ST_ permutation tests performed using Arlequin [Excoffier et al., 2005]*,* and randomization Monte Carlo simulation tests performed using macros written using Visual Basic for Excel. Sliding window nucleotide divergence analysis was performed using DnaSP 4.0 [Rozas et al., 2003], with bootstrap tests for heterogeneity performed using Excel. Tree and network generation was performed using Splitstree 4.0 [Huson and Bryant, 2006].

The genome-wide scan for single nucleotide polymorphisms SNPs associating with beta-defensin copy number and previously published *RHD* copy number [McCarroll et al., 2008] was performed on the founder CEU individuals (*n* = 60, 2.3 million SNPs), founder YRI individuals (*n* = 60, 2.6 million SNPs), and unrelated JPT/CHB samples (*n* = 90, 4 million SNPs) in PLINK 1.07 by linear regression under an additive model [Purcell et al., 2007]. *P*-Values were calculated in PLINK 1.07 by the Wald test and adjusted for multiple testing by a Bonferroni correction. Whole-genome SNP genotypes were downloaded from HapMap (release 23) and regional SNP association plots were generated. Pairwise LD (*r*^2^) between the selected “lead” SNP (red diamond) and neighboring HapMap SNPs were calculated using ssSNPer with the method described (http://gump.qimr.edu.au/general/daleN/ssSNPer/). CNV positions were taken from published data [Conrad et al., 2009], and all genome coordinates were based on hg18 (NCBI Build 36).

### Reporter Constructs and Transfection of Cell Lines

The pGL-promoter vector (Promega, Madison, WI) harbors the SV40 promoter that was excised using *Bgl*II and *Hind*III restriction enzymes (New England Biolabs). A 1.9-kb region of the *DEFB103* promoter region was amplified from BAC DNA clones H292 SCb-497j4 (clade II sequence, accession AF202031.5), RP11-1195F20 (clade IB sequence, accession AC130365.5), and RP11-287P18 (clade IA sequence, accession AC130360.4) (BACPAC Resources Center) by PCR using primers containing *Bgl*II/*Hind*III restriction sites (generated using primers 5′-GTCAAGATCTCAGCACCCATCCCACCCAC-3′, 5′-GTCAAAGCTTATGCTAGGCTTCACCCCAC-3′) and cloned into the digested pGL3 vector. Clones containing inserts were characterized by capillary sequencing using the vector specific primers RVprimer3 and GLprimer2. The pGL3-Basic vector and pGL-promoter vector were used as negative and positive controls, respectively.

HaCaT (program U-020) cell lines were transfected by nucleofection using the Ingenio™ Electroporation Kit for Lonza-amaxa Nucleofector devices according to the manufacturer's protocol (Mirus, Madison WI). Briefly, 1 × 10^6^ cells were cotransfected with 3 µg of the pGL3 plasmids and 300 ng of the pRL-TK Vector. Transfected cells were either unstimulated or cultured in media (DMEM supplemented with 10% fetal calf serum [FCS]) containing 100 ng/ml IFN-γ or 10 µg/ml lipopolysaccharide. After 24 hr the cells were washed in phosphate-buffered saline, lysed, and harvested by using 500 µl of Glo lysis buffer (Promega) per well. Firefly luciferase activity from the pGL3 reporter vectors and Renilla luciferase activity were determined using the Dual-Glo® Luciferase Assay System (Promega) according to the manufacturer's instructions. Luciferase values were recorded on a FLUOstar Omega microplate reader. The experiment was performed on at least three separate occasions, each time in triplicate, with the data representing a total of at least nine separate measurements. Promoter activity was normalized to the activity of the internal Renilla luciferase control.

## Results

To investigate the evolutionary history of the complex beta-defensin CNV, we compared this region in humans and chimpanzees. In chimpanzees, there is some evidence from array-CGH studies that the orthologous region shows CNV [Marques-Bonet et al., 2009; Perry et al., 2008], but the size of the array-CGH probes and the uncertainty, in paralogous regions, of the genome assembly prompted us to examine the locus in more detail. We developed a combined assay for analysis of chimpanzee beta-defensin copy number using both the PRT [Armour et al., 2007], which by comparing orthologous sequences was predicted to work on chimpanzee DNA, and a short tandem repeat assay within an intron of the *SPAG11* gene. Copy numbers of 4, 5, and 6 were observed, confirming CNV of this region in chimpanzees in a similar range to humans (Supp. Table S2), and highlighting the fact that the one assembled copy of this region in the chimpanzee genome assembly (CGSC 2.1/panTro2) is likely to be an error.

**Figure 1:**
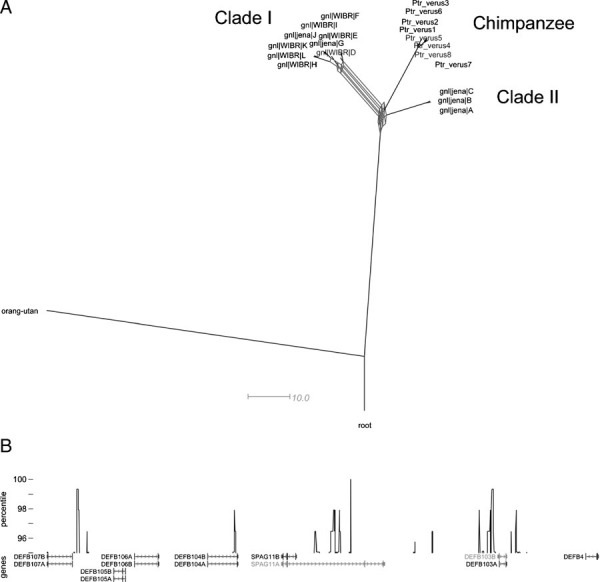
Evolutionary analysis of assembled beta-defensin copy number blocks. **A:** Recombination network of DNA sequences from a 3.2-kb region within the beta-defensin CNV block. The scale indicates number of nucleotide differences. **B:** Regions of the beta-defensin copy number repeat showing unusually high divergence of clade II. Peaks indicate regions that showed a divergence/diversity ratio in the top 5%.

To examine the evolutionary relationship between chimpanzee and human beta-defensin copies in more detail, we generated sequences of a random 3.2-kb region near *DEFB4* in chimpanzees, present as a single copy per block, using paralogue-specific PCR amplification from two chimpanzees (*Pan troglodytes verus*) each carrying four blocks, and also identified seven different unique orthologous sequences in humans from 12 previously sequenced BACs. A network of these sequences was generated; one clade containing nine human sequences (clade I) showed reticulations reflecting a history of gene conversion and recombination between these sequences (Fig. 1A). However, three sequences form a separate clade (clade II) with no evidence of recombination with clade I, at least in this 3.2 kb region. The clade II sequences are all identical to each other and are likely to be derived from a single copy sequence represented in three independent BACs sampling the same genomic region. The 3.2-kb *DEFB4*-linked region sequences thus led to the identification of sequenced BACs containing either clade I or II sequences that were used in subsequent investigations.

We compared the similarity of the clade II sequence with the two sequences representing clade I assembled in the human reference genome (hg18) across 105 kb of the beta-defensin block. Using a sliding window approach, the frequency of nucleotide differences between clade I and clade II was compared with the frequency of nucleotide differences within clade I. This is analogous to the widely used HKA test, which compares between-species differences to within-species diversity [Hudson et al., 1987]. Overall, the diversity between the clades was low (around 0.3%), but the pattern of clade I–clade II divergence was significantly heterogeneous across the region (*P*<2.2 × 10^−16^, runs test), with the top 5% of values clustering around, but not within, the exons of particular genes (Fig. 1B). This suggests either an unusually rapid sequence divergence, or absence of the homogenizing effect of gene conversion, between clades in potential regulatory regions. Importantly, there is only one protein-coding nucleotide change between the assembled clade I and clade II sequences (rs2740707 in *SPAG11*), suggesting that differences in beta-defensin protein sequences between clade I and clade II are not responsible for our observations.

**Figure 2:**
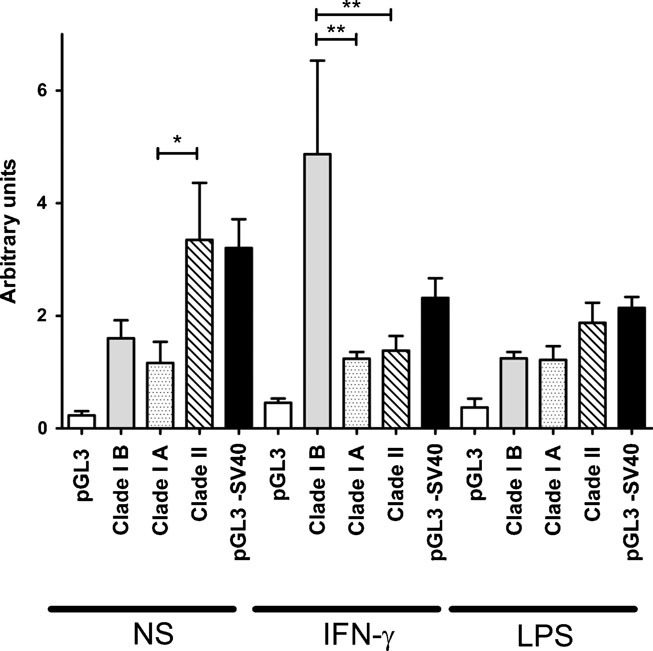
Characterization of *DEFB103* promoters from different clades. Promoter efficiency of 1.9 kb upstream region of *DEFB103* in HaCat keratinocyte epithelial cell lines, with pGL3 empty vector as negative control, and pGL3 with SV40 enhancer as a positive control, using a luciferase reporter gene assay. Three promoters, two from clade I corresponding to the two sequences assembled in hg18 human genome, and one from clade II, were tested under three conditions: nonstimulated (NS), interferon-gamma stimulation (IFN-gamma) and *Escherichia coli* lipopolysacharride stimulation (LPS). Statistically significant differences in expression between clades are shown, after correction for multiple comparisons (^*^P<0.05, ^**^P<0.01).

The lack of coding variation between clades I and II, and the sequence divergence between copies only at putative regulatory regions suggests natural selection is acting on gene expression. To test this, we analyzed the expression of the *DEFB103* gene, which encodes human beta-defensin 3 (hbd-3), a protein secreted by epithelia with broad-spectrum antimicrobial and antinflammatory activities, and which shows the strongest clade I–clade II divergence immediately upstream of exon 1. A 1.9-kb section of the immediate upstream region from two clade I BACs (clade IA and IB, representing the two sequences assembled in the hg18 human genome assembly) and the same 1.9-kb region from a clade II BAC were cloned into luciferase reporter vectors and transiently transfected into the immortalized keratinocyte epithelial cell line HaCat. The clade II sequence drove a twofold higher constitutive expression level of the reporter gene compared to the clade I sequences, demonstrating that sequence changes identified are responsible for variation in gene expression (Fig. 2). Sequence differences between the two clade I sequences also influence the response to interferon-γ, although there is no evidence that these have been subject to natural selection. Because there are no sequence changes that are unique to clade IB versus IA and II, the variable response to interferon-γ is likely to be an effect of more than one sequence change.

**Figure 3:**
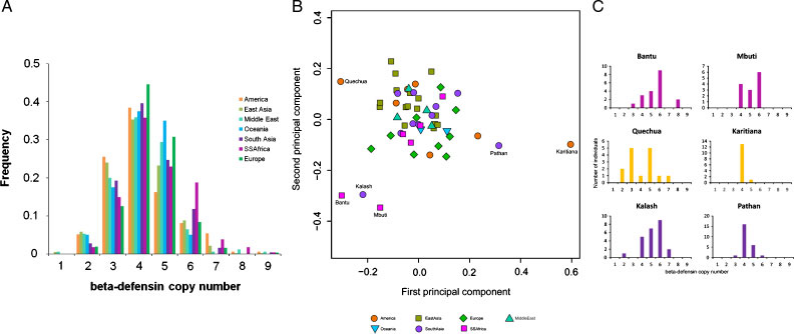
Principal component analysis of global variation of beta-defensin diploid copy number. **A:** Frequency distribution of worldwide beta-defensin copy number. **B:** Biplot of the first two principal components of beta-defensin copy number variation data, representing 76% of the total variation in the dataset. Outlier populations are highlighted. **C:** Frequency distributions of beta-defesin copy number in outlier populations.

We reasoned that if natural selection were acting on *DEFB103* gene expression, we might expect to see population-specific copy number distributions of clade I and clade II copy number. Initially, we typed 2015 individuals from 68 worldwide populations for total diploid beta-defensin copy number using PRT, and found diploid copy numbers between 1 and 9, with a modal copy number of 4, consistent with previous studies [Groth et al., 2008; Hollox et al., 2003, 2005] (Fig. 3A, Supp. Table S3). Nested analysis of variance showed that 96% of the observed copy number variation occurred within populations, with 2.2% between populations (*P* = 0.002) and 1.8% between continental regions (*P* = 0.01). To dissect the variation between populations, we plotted the first two principal components of the diploid copy number frequencies for each population (Fig. 3A). Neither regional grouping nor any pattern reflecting the range expansion out of Africa around 60,000 years ago was found. Instead, most populations cluster with no apparent geographical structure but with several outliers: Bantu, Mbuti, Quechua, Karitiana, Pathan, and Kalash (Fig. 3B). These appear to reflect a significantly different frequency for a particular copy number diplotype, either four-copy or six-copy, likely reflecting a real difference in two-copy and three-copy allele frequencies (Table 1). This effect is likely to be due to recent population processes such as genetic drift, and the two populations showing the most significant differences (Karitiana and Kalash) are known to be population isolates that have experienced genetic bottlenecks. Natural selection or genetic drift may be responsible for the pattern in the other outlier populations; nevertheless, given the importance of the beta-defensin genes in innate immunity, we speculate that these differences may have an effect on the susceptibility of the population to certain diseases.

**1:** Outlier Populations in Copy Number Distribution

Population	Copy number frequency difference	Significance (*P*)
Karitiana	4 copy increase	<0.0001
Kalash	6 copy increase	0.0024
Bantu	6 copy increase	0.0061
Quechua	4 copy decrease	0.0071
Pashan	4 copy increase	0.0086
Mbuti	6 copy increase	0.0271

Outliers were identified visually from principal component analysis (Fig. 3B) and particular changes in copy number frequency identify. Significance was assessed by a Monte Carlo randomization test against the null hypothesis that the difference is due to random sampling from the larger continental population.

**Figure 4:**
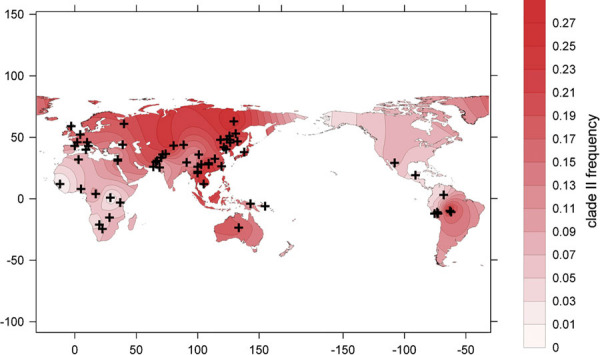
Global distribution map of clade II copy frequency. Frequency of clade II copies is shown as a contour map with frequency intervals colored according to the legend on the right of the map, and sampling locations shown as crosses (see also Supp. Table S1).

To determine the copy number of clade II blocks, we typed the rs2737902 multisite variant in the *DEFB103* promoter on a subset of samples (1,759 individuals, 67 populations). Its nonancestral type is a marker of clade II and is predicted to generate a GATA-1 binding site. Clade II block frequency, as a proportion of total copy number, showed a cline with the highest frequency in East Asia and lowest frequency in Africa (Table 2, Supp. Table S4, and Fig. 4). There was no association with a particular beta-defensin copy number beyond a positive correlation between clade II copy number and beta-defensin total copy number simply due to increased sampling (*P* = 0.3, χ^2^ test). Permutation tests confirm a significant difference (*P*<0.02) in rs2737902 diplotype frequencies in East Asia compared to other geographical regions, except Oceania. Because clade II carries the derived sequence and clade I the ancestral, this geographical pattern suggests that clade II rose to significant frequencies recently after the out-of-Africa range expansion.

**Table 2:** Frequency of Clade II Copies in Different Continental Groups

Continent	Number of individuals	Clade I copy count	Clade II copy count	Clade II frequency
Sub-Saharan Africa	268	1,174	66	0.05
America	332	1,256	128	0.09
Central-South Asia	230	839	158	0.16
East Asia	515	1,676	458	0.21
Europe	206	769	132	0.15

## Discussion

We have identified two types of human beta-defensin CNV block (clade I and II) that are distinguished by high sequence divergence at noncoding regulatory regions. Previously, it has been shown that the beta-defensin CNV is present at two loci (distal and proximal) separated by ∼5 Mb of single copy sequence [Abu Bakar et al., 2009]. This suggests a potential association between physical position and CNV block type. Testing this association is challenging, because we only know the physical location of the beta-defensin CNV blocks in two CEPH families. We tested one family for CNV block type and did not find a perfect association of clade I/clade II with CNV physical position. A subtler effect cannot be ruled out—for example, clade II copies occurring polymorphically only at the distal CNV location—but we do not have enough samples of known physical CNV position to test this thoroughly, as yet. Similarly, the relationship between type of beta-defensin CNV block and polymorphic inversion awaits a large cohort typed for the polymorphic inversion.

We show that clade II copies are at a significantly higher frequency in East Asians compared to other populations. Could this effect be due to hitchhiking of clade II sequences on haplotypes carrying another beneficial allele? We found no association genomewide of particular SNP alleles with total beta-defensin copy number or clade II copy number (Supp. Fig. S4a–d), suggesting that the beta-defensin CNV does not affect, nor is affected by, evolutionary processes at other linked loci by genetic hitchhiking effects. This observation, together with the absence of a signal at the beta-defensin CNV of the Out-of-Africa range expansion and a high copy number mutation rate of 0.7% [Abu Bakar et al., 2009], supports a model where signals of population movement and linkage disequilibrium of copy number with flanking SNPs are rapidly erased from beta-defensin CNV diversity. It follows that any population-specific variation that we see is a result of recent selective or demographic events.

Distinguishing effects of natural selection from genetic drift is particularly challenging for complex regions because it is not possible to compare the observed pattern of variation to a predicted pattern based on the standard neutral model. Recent work has begun to construct a framework for neutral modelling of fixed duplicate genes [Innan and Kondrashov, 2010; Thornton, 2007], and polymorphically duplicated regions, but as yet more complex CNV regions are not described. This may be a consequence with the extra parameters required to model variation, and the consequent loss of predictive power of such models. The result is a lack of well-validated tools that allow us to detect departures from a neutral model. However, based on the neutral assumption that the different blocks are evolving at the same rate between blocks and across the block, we used a HKA-like test that tests for departures from an equal rate of sequence divergence across the three different beta-defensin blocks, and a runs test that tests for significant heterogeneity in the rate of sequence divergence across the block.

The evidence, presented here, that selection has operated on clade II sequence, that clade II encodes higher expression of *DEFB103* in epithelial cells, and that clade II is at highest frequency in East Asia prompted us to look for a selective explanation for our observations. hbd-3 protein acts against several microbes that colonize and infect epithelia, including *Staphylococcus aureus* and *Candida albicans* [Harder et al., 2001], and viruses such as HIV-1 [Sun et al., 2005]. Additionally, hbd-3 inhibits influenza viral fusion with epithelial cells by forming a protective barrier of immobilized surface proteins [Leikina et al., 2005], so it is likely that higher constitutive expression levels provide increased resistance to influenza infection. Further functional experiments relating *DEFB103* genomic variation to hbd-3 expression levels on epithelia and variability to infection are necessary to conclusively establish this link. Over the past 10,000 years East Asia has developed a characteristic agriculture, combining dense human population settlements and intensive pig-rearing with standing water for wildfowl. Because of this environment, the region is regarded as the source of many historical influenza epidemics [Wolfe et al., 2007]. Thus, it is likely that this region suffered an extensive burden of influenza in the past, and this is a plausible selective explanation for our observations. Both Karitiana and Surui in South America show high frequencies of clade II copies, which distinguishes them from the seven other Amerindian populations studied, and could be due to genetic bottlenecks, recent influenza mortality, or a combination of both.

Selection for a higher level of expression is consistent with the lack of coding sequence divergence between paralogues, as the selected trait is increased or decreased levels of the same protein. In this region of the genome, CNV affects gene dosage of all genes in the block, whereas single nucleotide variation can affect the expression of just one gene. Although both forms of variation are present in the population, an increase of hbd-3 protein level may have been most advantageous. Indeed, increased expression of the other beta-defensin genes may even have been disadvantageous, favoring the spread of a sequence variant that increases hbd3 levels specifically. This model should prompt further studies on the evolutionary and functional relevance of sequence variation within CNV for the beta-defensin CNV [Groth et al., 2010; Huse et al., 2008; Taudien et al., 2010], and for other CNV regions. Selection on gene expression levels of CNV genes is consistent both with the long-term maintenance of CNV and maintenance of nucleotide variation within promoter regions.
