# Supplementary material for: A Worldwide Analysis of Beta-Defensin Copy Number Variation Suggests Recent Selection of a High-Expressing DEFB103 Gene Copy in East Asia
Source: Hum Mutat. 2011 Mar 8;32(7):743–50. doi: 10.1002/humu.21491 (PMC3263423; doi:10.1002/humu.21491)
Supplement: Supplementary file 1 [file humu0032-0743-SD1.pdf]

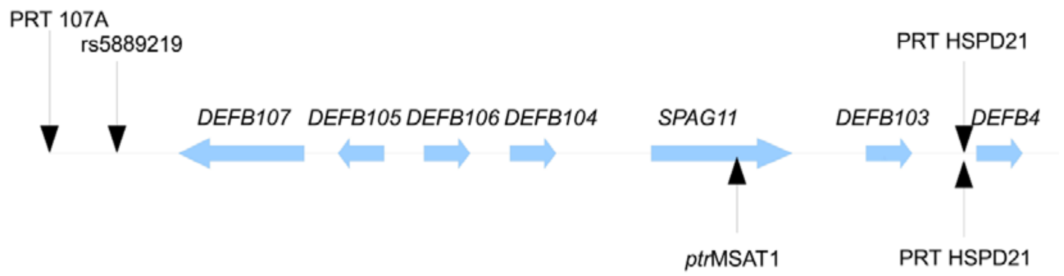

Supp. Figure S1. Copy number assays and locations. This shows a single assembled beta-defensin region, which is copy number variable *en bloc*. The three assays comprising the triplex PRT approach (Aldhous et al., 2010) are shown above the genes, and consist of two PRTs with reference loci on other chromosomes (PRT107A, HSPD21), and a variable length indel (rs5899219). Below the genes are shown the assays used to measure chimpanzee copy number.

Supp. Figure S2. Quality analysis of beta-defensin copy number data

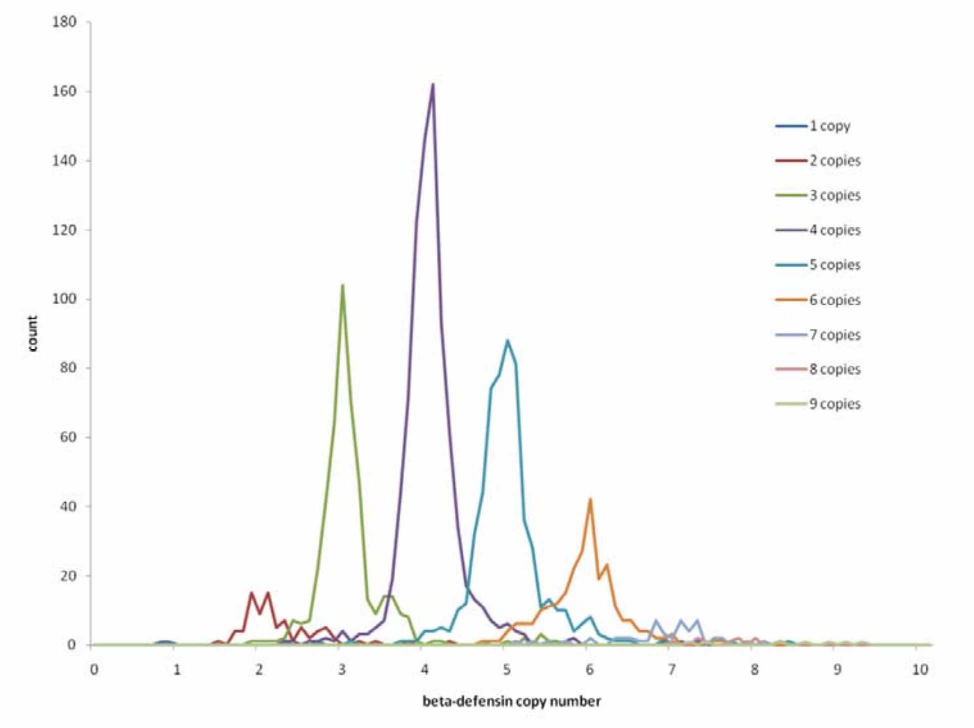

Supp. Figure S2a. A histogram of the mean raw value of the triplex PRT assay for all samples and controls tested in this project, divided according to final copy number, as determined by the maximum-likelihood. This shows the clear clustering of raw values about integer copy numbers, and also how the maximum-likelihood approach can distinguish integer copy number between the peaks by taking the different errors of the three PRT assays into account. It can be seen that a simple binning approach based on the raw copy number would likely misclassify a proportion of samples with raw copy numbers between integer values.

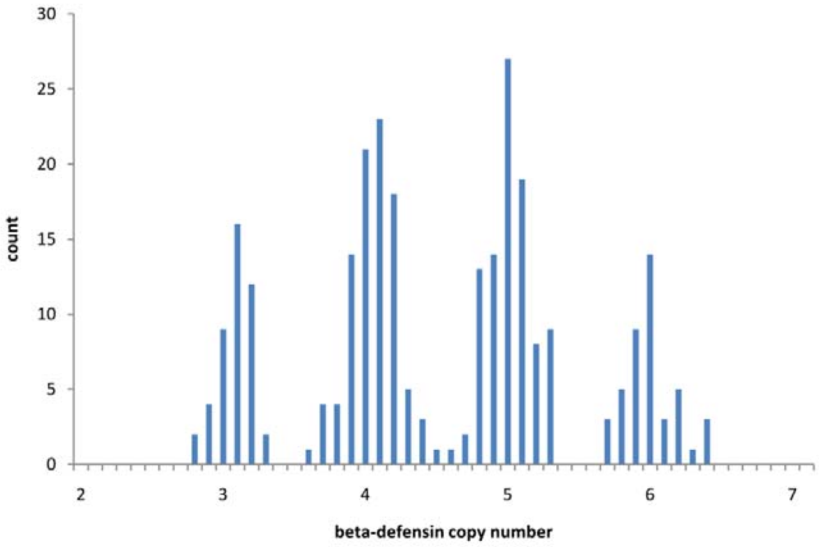

Supp. Figure S2b. A histogram of the mean raw value of the triplex PRT assay for 280 controls included in every copy number typing experiment. It represents repeat testing of six DNA samples, available from the European Collection of Cell Cultures, CO088 4 copies, CO207 5 copies, CO849 6 copies, CO913 3 copies, CO940 4 copies, CO969 5 copies.

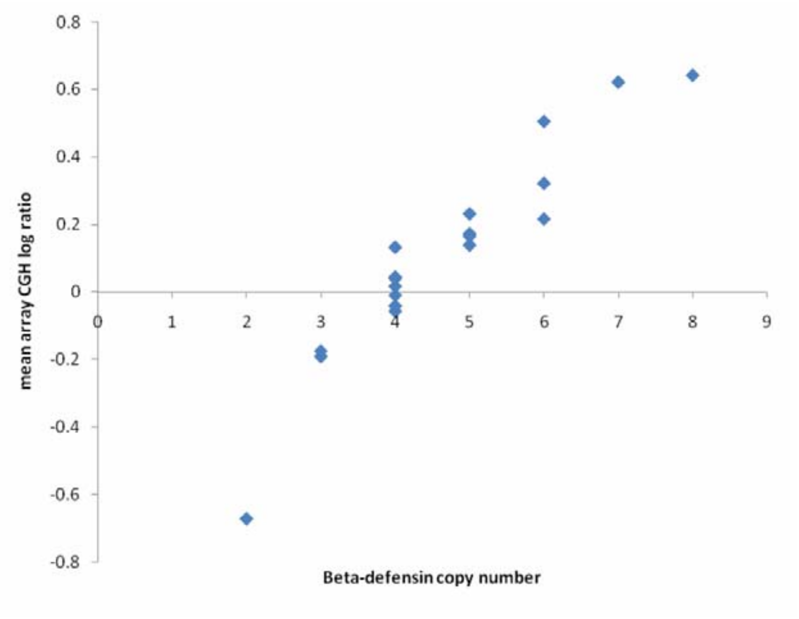

Supp. Figure S2c. Comparison of raw whole-genome tiling array-CGH data spanning the beta-defensin segmental duplication and PRT-based beta-defensin copy number for 19 HapMap samples. Array-CGH data is a mean of 5117 oligonucleotide probes (hg18 coordinates chr8:7121228-7431058, available from the Wellcome Trust Sanger Institute website).

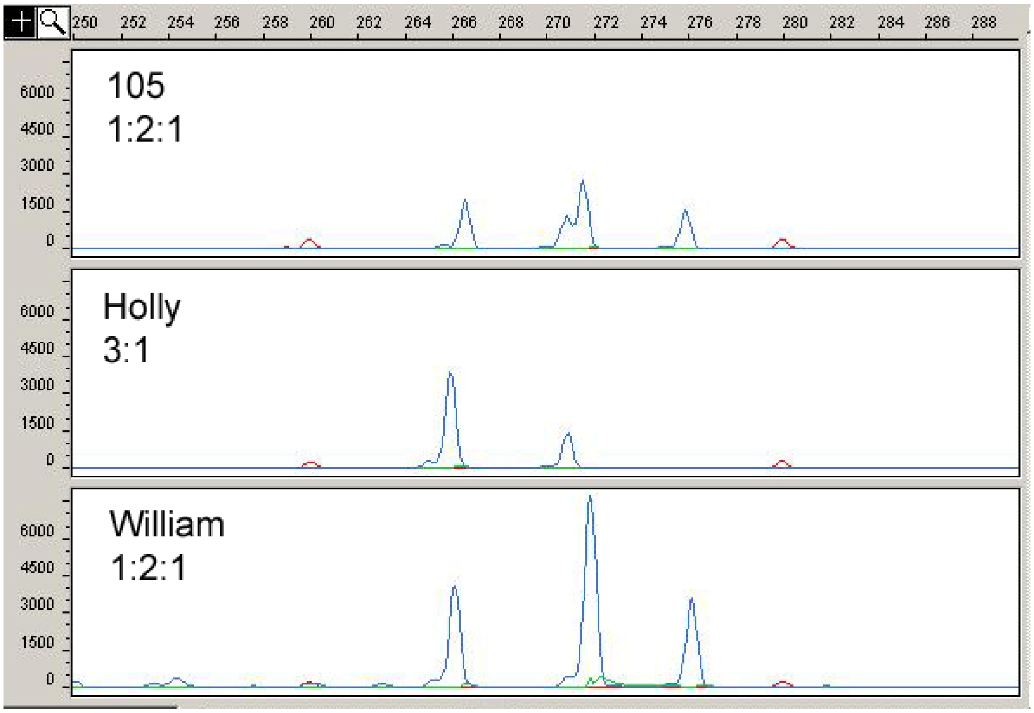

Supp. Figure S3. STR assay on chimpanzee samples. Three examples of the STR assay on chimpanzee DNA, showing electropherograms of fluorescently-labelled PCR products amplified across the STR. The ratios of the areas under each peak are shown, giving information about diploid copy number at this locus.

Supp. Figure S4a,b,c. Absence of association of beta-defensin copy number with neighbouring SNPs

Legend: This shows fine-scale association map of the 8p23.1 and flanking regions, following a genomewide association analysis on HapMap samples and beta-defensin diploid copy number. REPP, and REPD, the olfactory repeat regions within which the beta-defensin copy number blocks are embedded are annotated (Giglio et al., 2001; Giglio et al., 2001; Sugawara et al., 2003; Abu Bakar et al., 2009). The highlighted SNP (rs3177011) was selected randomly within the beta-defensin repeat region and is in the *SPAG11* gene. Individual SNPs are plotted as diamonds, coloured according to the linkage disequilibrium with rs3177011, with red indicating $0.8<r^2<1$, orange indicating $0.5<r^2<0.8$ and yellow indicating $0.2<r^2<0.5$. The y-axis shows significance of association after Bonferroni correction for multiple tests. Association analysis using clade II copy number shows exactly the same lack of association after Bonferroni correction.

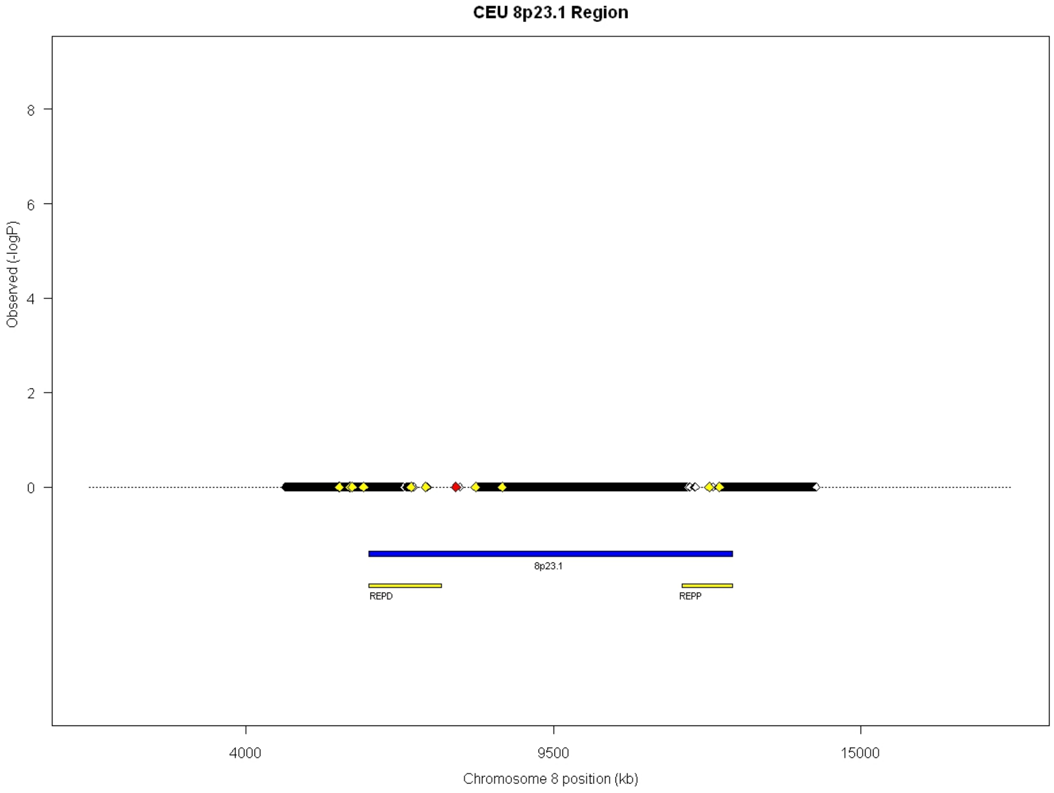

Supp. Figure S4a.

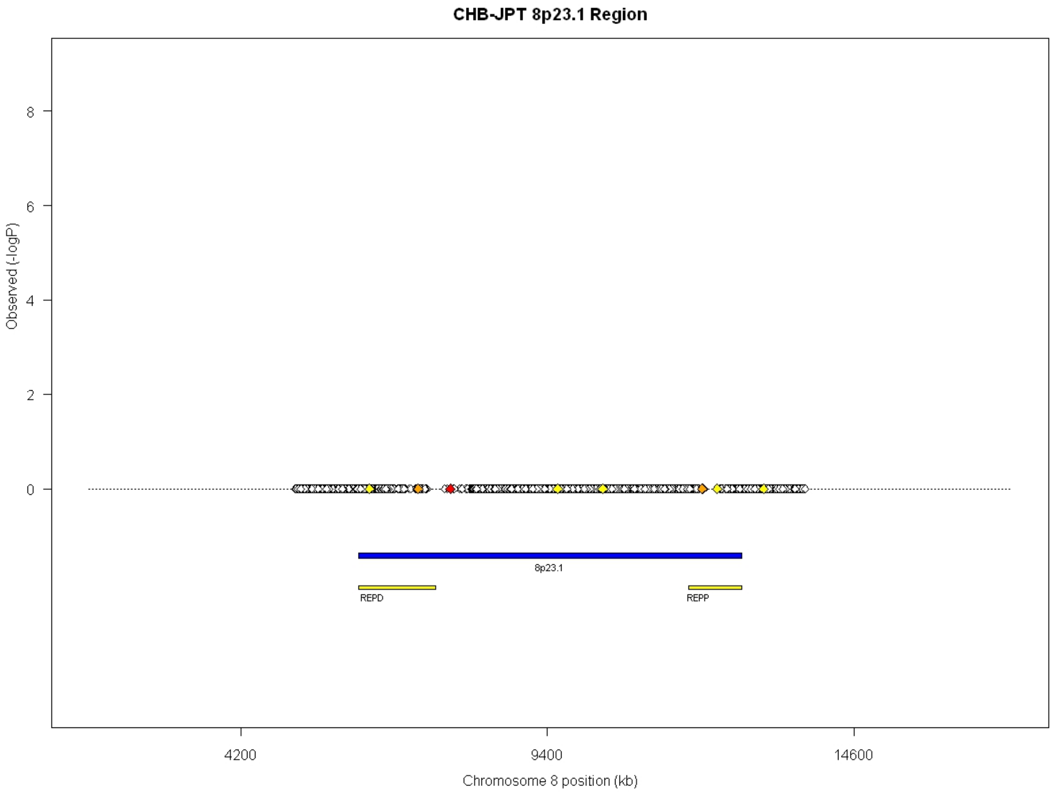

Supp. Figure S4b.

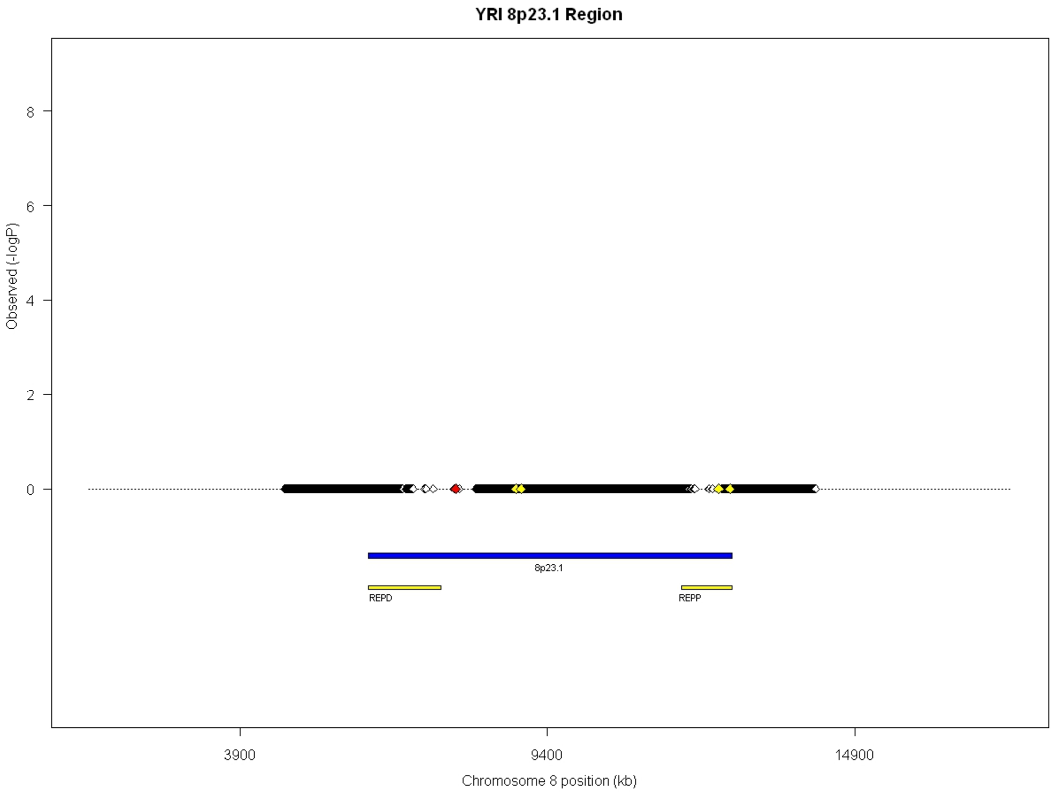

Supp. Figure S4c.

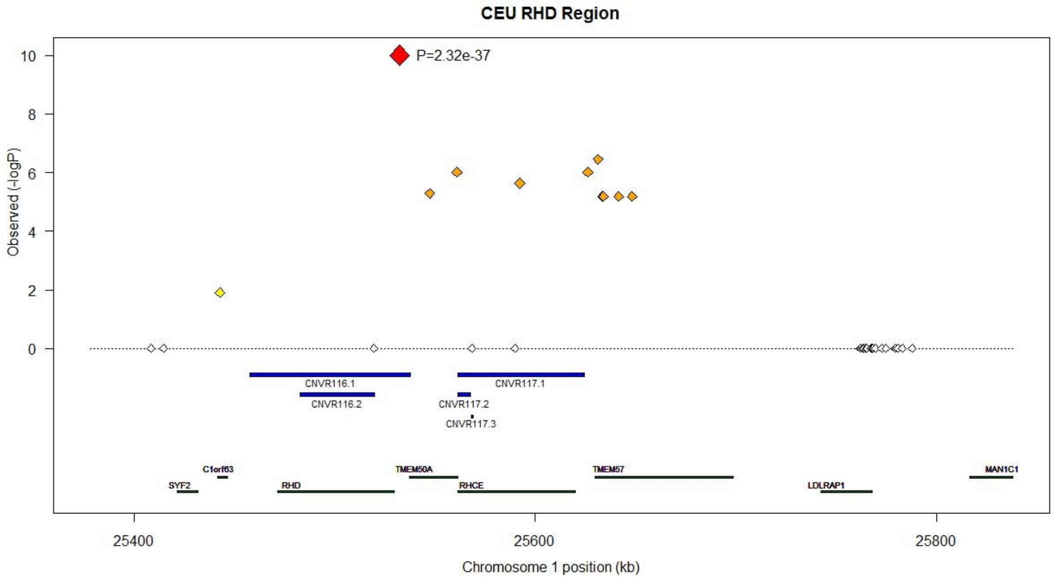

Supp. Figure S4d. As a positive control, we performed genome-wide association using previously published copy number of the *RHD* gene, a simple deletion. A clear signature of association with *RHD* copy number is seen in the SNPs around the *RHD* gene.

Supp. Table S1. Geographical coordinates, sample sizes and mean copy number of individual populations

Population	Location	Mean copy number	Number of samples for copy number analysis	Number of samples for clade II analysis	Region
**Adygei**	44, 39	4.5	17	17	Europe
**Ashaninka**	-11.1, -73.4	4.1	142	141	America
**Balochi**	31, 67	4.5	24	24	SouthAsia
**Bantu N.E.**	-3, 37	5.5	11	11	SSAfrica
**Bantu S.E and S.W**	-24.5, 22.5	5.5	8	8	SSAfrica
**Bedouin**	31, 35	4.1	47	47	MiddleEast
**Biaka Pygmies**	4, 17	4.1	27	24	SSAfrica
**Brahui**	30, 66	4.4	25	25	SouthAsia
**Burusho**	36.5, 74	4	25	21	SouthAsia
**Cambodian**	12, 105	4.7	9	9	EastAsia
**CEU_HapMap**	52.2, 4.5 *	4.4	60	49	Europe
**Chinese-Korean**	42.0, 121.4	4.4	21	17	EastAsia
**Colombian**	3, -68	3.9	7	6	America
**Dai Chinese**	21, 100	4.8	10	9	EastAsia
**Daur Chinese**	48.0, 118.6	4	27	23	EastAsia
**Druze**	32, 35	4.2	44	44	MiddleEast
**Ewenki Chinese**	48.0, 118.6	4.4	19	16	EastAsia
**French**	46, 2	4.4	28	27	Europe
**French Basque**	43, 0	3.9	24	24	Europe
**Han Harbin**	45.7, 126.6	4.4	35	32	EastAsia
**Han Yili**	43.3, 80.5	4	25	22	EastAsia
**HanChinese HGDP+HapMap**	32.5, 114	4	90	59	EastAsia
**Han-Mongolia**	48, 126.3	4.3	18	12	EastAsia
**Hazara**	33.5, 70	3.9	23	23	SouthAsia
**Hezhe-Chinese**	46.7, 132.5	3.8	44	34	EastAsia
**Japanese HGDP+HapMap**	38, 138	4.3	73	69	EastAsia
**Kalash**	36, 71.5	5.2	24	23	SouthAsia
**Karitiana**	-10, -63	4.1	14	15	America
**Lahu-Chinese**	22, 100	3.8	8	8	EastAsia
**Mainland UK**	54,2 **	4.4	94	nt	Europe
**Makrani**	26, 64	4.5	25	24	SouthAsia
**Manchu**	40.2, 123.2	3.9	31	27	EastAsia
**Mandenka**	12, -12	4.4	24	22	SSAfrica
**Maya**	19, -91	4.3	22	19	America
**Mbuti Pygmies**	1, 29	5.2	13	12	SSAfrica
**Miaozu-Chinese**	28, 109	4	10	9	EastAsia

Population	Location	Mean copy number	Number of samples for copy number analysis	Number of samples for clade II analysis	Region
**Mongolian**	51.0, 126.0	4.1	34	24	EastAsia
**Monte Carmelo**	-12.1, -72.6	3.9	22	22	America
**Mozabite**	32, 3	4	29	29	MiddleEast
**Nan Melanesian**	-6, 155	4.4	13	13	Oceania
**Native Australian**	-23.4, 133.5	3.9	10	10	Oceania
**Naxi Chinese**	26, 100	3.9	9	9	EastAsia
**North Italian**	46, 10	4.7	13	13	Europe
**Orcadian**	59, -3	4.5	15	15	Europe
**Oroqen Chinese**	53.0, 131.0	4	28	26	EastAsia
**Palestinian**	32, 35	4.4	50	50	MiddleEast
**Papuan**	-4, 143	4.2	17	17	Oceania
**Pathan**	33.5, 70.5	4.3	24	24	SouthAsia
**Pima**	29, -108	4.6	14	14	America
**Quechua**	-12.2, -75.5	4.1	15	15	America
**Russian**	61, 40	4.6	25	25	Europe
**San**	-21, 20	4.5	6	6	SSAfrica
**Sardinian**	40, 9	4.2	28	28	Europe
**She-Chinese**	26.4, 119.5	3.6	10	10	EastAsia
**Shimaa**	-12.3, -73.1	4.4	88	89	America
**Sindhi**	25.5, 69	4	23	23	SouthAsia
**Surui**	-11, -62	4.4	9	9	America
**Tibet**	29.6, 91.1	3.5	24	14	EastAsia
**Tu Chinese**	36, 101	4	10	10	EastAsia
**Tuija Chinese**	29, 109	4.3	10	8	EastAsia
**Tuscan**	43, 11	4	8	8	Europe
**Uygur Urumqi**	43.8, 88.6	4.1	30	20	SouthAsia
**Uygur Yili**	43.3, 80.5	4.2	32	23	SouthAsia
**Xibe**	43.3, 80.5	4.1	37	33	EastAsia
**Yakut**	63, 129.5	4.5	25	25	EastAsia
**Yizu**	28, 103	4.3	10	10	EastAsia
**Yoruba HGDP+HapMap**	8, 5	4.5	79	79	SSAfrica
**Zambian**	-15.2, 28.2	4.7	120	106	SSAfrica

* The CEU_HapMap samples were collected from individuals of European Ancestry from Utah, USA. Whole genome SNP analysis confirms that they are of north-western European ancestry, with a mean location of present-day Netherlands. The coordinates given here, and used, are for the city of Rotterdam.
** The mainland UK population are from individuals across the United Kingdom. The coordinates given here correspond to the geographical centre of the UK.
SSAfrica = Sub-Saharan Africa; nt = not tested

Supp. Table S2. Beta-defensin copy number estimates of chimpanzees

Individual	Species	ptrMSAT1 variants	ptrMSAT1 variant ratio	ptrMSAT1 copy number	HSPD21 PRT	Copy number
**EB176**	*P.troglodytes*	262,272,276	1:3:1	4.7-5.0	4.4-4.9	5
**105**	*P.troglodytes verus*	266,272,276	1:2:1	4.1-4.3	3.7-4.1	4
**75**	*P.troglodytes verus*	266,272	1:1	2.0-2.0	4.0-4.4	4
**68**	*P.troglodytes verus*	252,268	1:3	3.7	3.4-3.6	4
**27**	*P.troglodytes verus*	266,272	1:3	3.9-4.3	3.4-4.0	4
**12**	*P.troglodytes verus*	266,270	1:1	2.0-2.0	3.9-4.1	4
**Kip**	*P.troglodytes troglodytes*	262,266,272	1:1:5	4.1-5.9	n.t.	5
**Mongo**	*P.troglodytes troglodytes*	266,272	1:3	3.6-3.9	3.7-4.5	4
***Sam**	*P.troglodytes troglodytes*	262,266,272	5:1:1	6.9-7.0	5.3-6.4	6 or 7
**Holly**	*P.troglodytes troglodytes*	266,272	3:1	4.0-4.1	2.6-4.5	4
**Vicky**	*P.troglodytes troglodytes*	262,266,272	1:1:2	4.3-4.3	6.0-6.4	Unclear
**Peter**	*P.troglodytes troglodytes*	262,272	1:1	1.6-2.1	4.4-4.8	4
***Choppers**	*P.troglodytes troglodytes*	266,272	1.5:1	5.0-5.3	n.t.	5
**Elly**	*P.troglodytes troglodytes*	262,266,272	1:1:1	3.0-3.0	5.4-6.5	6
***Noddy**	*P.troglodytes troglodytes*	266,277	1:1	2.0-2.2	3.9-4.3	4
**Ricky**	*P.troglodytes troglodytes*	262,266	3:1	4.1-4.2	3.4-4.0	4
**William**	*P.troglodytes troglodytes*	266,272,275	1:2:1	3.8-4.4	5.6-6.6	Unclear

HSPD21 PRT reports absolute diploid copy number, and the STR assay reports a number of which the copy number can be any multiple; for example a value of 2 can represent 2, 4, 6 or indeed any other multiple of two. EB176 is DNA derived from a chimpanzee lymphoblastoid cell line available from the European Collection of Cell Cultures. * wild-born

Supp. Table S3. Full copy number data, is available online as a separate Supporting Information Excel File.

Supp. Table S4. Frequency of clade I and clade II copies in different populations

Population	Number	Clade I Count	Clade II Count	Clade I Freq	Clade II Freq
Aboriginal Australian	10	32	7	0.82	0.18
Adygei	17	65	12	0.84	0.16
Ashaninka	141	542	28	0.95	0.05
Balochi	24	91	16	0.85	0.15
Bantu N.E.	11	58	3	0.95	0.05
Bantu S.E and S.W	8	41	3	0.93	0.07
Bantu (Combined)	19	99	6	0.94	0.06
Bedouin	47	161	32	0.83	0.17
Biaka Pygmies	24	86	10	0.90	0.10
Brahui	25	83	26	0.76	0.24
Burusho	21	66	17	0.80	0.20
Cambodian	9	31	11	0.74	0.26
CEU_HapMap	49	185	30	0.86	0.14
ChineseKorean	17	57	20	0.74	0.26
Colombian	6	20	1	0.95	0.05
Dai	9	34	9	0.79	0.21
Daur	23	71	22	0.76	0.24
Druze	44	168	15	0.92	0.08
Ewenki	16	53	15	0.78	0.22
French	27	101	21	0.83	0.17
French Basque	24	83	9	0.90	0.10
Han	24	79	21	0.79	0.21
Han_HapMap	35	124	17	0.88	0.12
HanHarbin	32	112	29	0.79	0.21
HanYili	22	69	21	0.77	0.23
Hazara	23	80	9	0.90	0.10
Hezhen	34	105	27	0.80	0.20
Japanese	26	73	32	0.70	0.30
JPT_HapMap	43	151	40	0.79	0.21
Kalash	23	97	25	0.80	0.20
Karitiana	15	50	20	0.71	0.29
Lahu	8	23	7	0.77	0.23
Makrani	24	90	16	0.85	0.15
Manchu	27	83	26	0.76	0.24
Mandenka	22	99	0	1.00	0.00
Maya	19	77	3	0.96	0.04

Population	Number	Clade I Count	Clade II Count	Clade I Freq	Clade II Freq
**Mbuti Pygmies**	12	61	0	1.00	0.00
**Miaozu**	9	26	9	0.74	0.26
**HanMongolia**	12	43	12	0.78	0.22
**Mongola**	24	73	27	0.73	0.27
**MonteCarmelo**	22	76	6	0.93	0.07
**Mozabite**	29	107	9	0.92	0.08
**Nan Melanesian**	13	47	10	0.82	0.18
**Naxi**	9	32	3	0.91	0.09
**North Italian**	13	51	10	0.84	0.16
**Orcadian**	15	54	14	0.79	0.21
**Oroqen**	26	80	22	0.78	0.22
**Palestinian**	50	191	31	0.86	0.14
**Papuan**	17	65	6	0.92	0.08
**Pathan**	24	93	10	0.90	0.10
**Pima**	14	59	5	0.92	0.08
**Quechua**	15	56	5	0.92	0.08
**Russian**	25	94	22	0.81	0.19
**San**	6	26	1	0.96	0.04
**Sardinian**	28	109	9	0.92	0.08
**She**	10	28	7	0.80	0.20
**Sindhi**	23	85	8	0.91	0.09
**Shimaa**	89	337	51	0.87	0.13
**Surui**	9	33	7	0.83	0.18
**Tibet**	14	41	6	0.87	0.13
**Tu**	10	35	5	0.88	0.13
**Tujia**	8	26	9	0.74	0.26
**Tuscan**	8	27	5	0.84	0.16
**UygurUrumqi**	20	66	13	0.84	0.16
**UygurYili**	23	88	18	0.83	0.17
**Xibo**	33	111	21	0.84	0.16
**Yakut**	25	82	31	0.73	0.27
**Yizu**	10	34	9	0.79	0.21
**Yoruba**	21	86	10	0.90	0.10
**YRI_HapMap**	58	252	12	0.95	0.05
**Zambian**	106	465	36	0.93	0.07
